# Comparison between Concentration and Immersion Based on EEG Analysis

**DOI:** 10.3390/s19071669

**Published:** 2019-04-08

**Authors:** Seokbeen Lim, Mina Yeo, Gilwon Yoon

**Affiliations:** 1 Department of Electronic Engineering, Graduate School, Seoul National University of Science and Technology, Seoul 01811, Korea; dandansurcle@seoultech.ac.kr (S.L.); mayeo@synex.co.kr (M.Y.); 2 Department of Electronic & IT Media Engineering, Seoul National University of Science and Technology, 232 Gongneung-ro, Nowon-gu, Seoul 01811, Korea

**Keywords:** electroencephalography, mental state, independent component analysis, concentration, immersion, computer game

## Abstract

Concentration and immersion belong to a similar mental state in which a person is preoccupied with a particular task. In this study, we investigated a possibility of diagnosing two mental states with a subtle difference. Concentration and immersion states were induced to analyze the electroencephalography (EEG) changes during these states. Thirty-two college students in their 20s participated in the study. For concentration, subjects were asked to focus on a red dot at the center of a white screen, and for immersion they were asked to focus on playing a computer game. Relative to rest, Alpha waves decreased during concentration and immersion. Relative to rest, Theta waves decreased at almost all channels during concentration and, on the other hand, increased at all channels during immersion. Beta waves increased during concentration and immersion in the frontal and occipital lobes, with a higher increase in immersion. In the temporal lobe, Beta waves decreased during concentration and increased during immersion. In the central region, Beta waves decreased during concentration and immersion, and the decrease during immersion was larger. Such evident differences between the EEG results for concentration and immersion can imply diagnostic capabilities of various other mental states.

## 1. Introduction

Electroencephalography (EEG) is one of the noninvasive methods for investigating brain activity. It is safe and easy to perform, and has the advantage that it can measure the change in brain activity in real time and for personal use [1]. The frequency bands of the EEG signals represent four different brain activities; the Delta band (0.5–4 Hz) for the state of deep sleep and waking, the Theta band (4–8 Hz) for the state of consciousness slips, creative inspiration, deep meditation, and arousal. The Alpha band (8–12 Hz) indicates the state of relaxed awareness without attention, and the Beta band (12–30 Hz) shows the state of the active thinking, active attention, and solving problems. The Gamma band (>30 Hz) is sometimes related with brain diseases [2].

Attention deficit hyperactive disorder sub-types in children have been identified using spectral analysis [3,4]. An important benefit of EEG is that it can be used for human–machine interfaces because it is cheap and equipment for EEG is easy to manufacture in small sizes [5,6,7]. Damasevicius et al. applied EEG for a biometric authentication method where EEG data from 42 subjects were used, achieving an Equal Error Rate of 0.024 [8]. In sports health, a wearable Internet of Things-based system has been used to record EEG. In this application, the removal of the movement artifacts was an important issue [9]. Other area of research has been in analyzing mental states such as rest, concentration and immersion. Most studies distinguish concentration from immersion from a social science viewpoint through questionnaires. 

Concentration and immersion appear to be similar mental states with a state of preoccupation with a particular object or task. However, there are some differences. Concentration is a state where information is selectively received, and it is related to the limit of the amount of information that can be processed at one time [10,11]. On the contrary, immersion causes a person to forget the surroundings or personal problems while being immersed in a particular object [12]. Jennett et al. defined cognitive involvement, real-world dissociation, emotional involvement, challenge, and control as the five elements of immersion and claimed that real-world dissociation is a common feature of concentration and immersion. However, the phenomenon of real-world dissociation manifests differently in concentration and immersion, and, as a result, immersion cannot be completely explained by the theory of concentration [13]. 

Regarding the changes in EEG results in the concentration and immersion states, Ray et al. suggested that the concentration state is associated with Alpha waves [14]. Lee noted that attention levels increased in the frontal lobes of archers before they bowed, which indicated that Beta waves were dominant relative to Alpha waves [15]. In the study of Rodrak and Wongsawat, the power ratio (Beta/Alpha) of Beta and Alpha waves was used as an index to determine the concentration state in a neurofeedback system for patients with attention deficit/hyperactivity disorder (ADHD) [5]. Srinivasan et al. suggested that the frontal and parietal cortices are activated during concentration using EEC and fMRI measurements [16]. Sung et al. and Ga et al. used the sum of sensorimotor rhythm (SMR) and mid-Beta (Mid-B) waves divided by Theta waves as an EEG index to determine the concentration state. Here, SMR and Mid-B waves are detailed classifications of Beta waves; the frequency bands of SMR, Mid-B, and high-Beta waves are 12–14.99 Hz, 15–19.99 Hz, and 20–30 Hz, respectively [17,18]. Engelke et al. assessed the quality of experience (QoE) through biosignals such as brain waves. High-level QoE included immersion, and Theta waves, which represented concentration decline, and Alpha waves, which represented increased relaxation, were used to evaluate QoE [19]. However, the number of subjects was five without meaningful statistical analysis. Myrden et al. described the Alpha band expressed in the larynx as a predictor of changes in attention levels [20]. In a study by F.J. Perales et al., the changes in EEG results while playing a video game were obtained for a young patient with cerebral palsy and for a control group. It was reported that the Alpha band decreased and the Beta band increased in the state of focused attention. The same phenomenon was observed in patients with cerebral palsy and in a control group, yet the control group had higher power values [21]. 

In this study, EEG was performed during rest, concentration, and while playing a computer game and data were preprocessed for spectrum analysis. For normal subjects, the rest, concentration, and immersion states were compared by deriving absolute power and an index. In particular, focus was given on whether concentration and immersion can be distinguished. EEG was performed at rest to distinguish between the concentration and immersion states during the game. As this study is not intended for patients with sleeping or brain diseases, we focused on Theta, Beta, and Alpha waves while excluding Delta and Gamma waves, used digital filter and independent component analysis (ICA), and removed interference signals and noise components such as EEG and electrooculography (EOG) signals to reduce the influence of noise. This study differs from the previous studies in that it finds an indicator that distinguishes between the immersion and concentration states during game playing using brain waves. Most of the previous work cited in the above had a limited number of subjects and it is difficult to conclude with meaningful statistical significance. This study aims to identify the differences between concentration and immersion in neurophysiology and to find indicators that can distinguish between concentration and immersion states.

## 2. Experimental Methods and Signal Processing

The study subjects consisted of 33 men and women in their 20s who had no neurological and psychiatric illnesses, and one subject was excluded from analysis owing to data error. The recruited subjects were adults who had played League of Legends for at least one month before participating in the experiment. Out of the 32 subjects, 29 were men (90.63%) and 3 were women (9.38%). The mean age was 25.78 years with a standard deviation of ±2.35. To set the number of subjects, we applied the paired t-test. The effect size was set to 0.5, power to 0.8, and significance level to 0.05. The calculation gave a sample size of 27 and it was appropriate since our subjects numbered 32. The experiments in this study were approved by Public Institutional Review Board Designated by Ministry of Health and Welfare, Korea.

Brain waves were measured using QEEG-8^TM^ (Laxtha Inc., Daejeon, Korea). There were 8 channels, and the Fp1, Fp2, T3, C3, C4, T4, O1, and O2 positions were measured based on the 10–20 system. The channels of the electrodes were numbered sequentially, starting from 1 for Fp1 to 8 for O2. A reference electrode was attached to the proximal cervical vertebrae of the 7th cervical vertebra, and a ground electrode was attached to the right mastoid. A disk electrode made of gold-plated brass was used. This electrode had a circle cup form and with a diameter of 10 mm. To reduce the impedance between the electrode and the skin, first skin surface was scrubbed using skin prepping gel (Nuprep Gel, Weaver and Company, Aurora, CO, USA) and conductive paste (Ten 20 conductive paste, Weaver and Company, Aurora, CO, USA) was applied. Then, the gauze was used to fix the electrode on the skin surface. The experiments were conducted in the order of rest, concentration, and immersion. 

Rest was measured in a relaxed state with minimal external stimuli. Subjects were presented with a blank white screen with a size of 420 mm × 300 mm on the 20-inch monitor to reduce visual stimulation in an environment with no noise. The concentration state was induced through visually sustained attention by presenting a white screen with a red dot in the middle. The screen size was the same as that used for the rest measurement, and the red dot was 10 mm in diameter. Subjects were asked to concentrate on the red dot for 5 min. After the concentration experiment, subjects were provided a resting time of approximately 5 min, and then, the immersion experiment was performed. Subjects played the computer game of Multiplayer Online Battle Arena (MOBA) genre (League of Legend, Riot Games Inc.) to induce immersion. This game was selected because 40.1% of computer game users play the genre of MOBA games and most users play LoL among them. The company estimated over 100 million active players each month in 2016. Subjects used the keyboard (TGK-8600, Lite-On Technology Corp., Hsinchu, Taiwan) and the mouse (JSCO noiseless JNL-006k, Shenzen, China) to control their game characters. During the immersion measurement, subjects were allowed to hear the sound of the game to increase the degree of immersion.

Laxtha Telescan^TM^ was used for data collection. This instrument includes a bandpass filter (0.6–46 Hz), and its sampling frequency is 512 Hz. The measurement time was 5 min each for rest and concentration. The time of the gameplay was not fixed. It depended on each player and ranged from approximately 20–40 min. During the gameplay, EEG data were acquired every 5 min. Assuming that the signal at the beginning of the measurement best reflects the state, the measurement of the first minute was used for the analysis of rest and concentration. However, in the case of immersion, as the game starts in earnest after 100 s owing to its characteristics, the data for 1 min after 100 s were analyzed.

EEG is easy to perform; however, it is considerably affected by noise. The largest noise is the EOG signals resulting from eye flickering or movement. In addition, there are electrocardiography (ECG) and electromyography (EMG) signals caused by forehead and jaw movement. EOG signals primarily have a low frequency of less than 4 Hz, which affects the Fp1 and Fp2 channels, and they are the most interfering signal for EEG measurement because of their large amplitude. The frequency of EMG signals is typically significantly higher than that of brain waves, and these signals are mostly removed during EEG signal processing. In addition, there are interfering signals from alternating current power noise and fluorescent light. Such noise may result in erroneous brain wave measurement results or poor performance in brain–computer interfaces [22].

**Figure 1:**
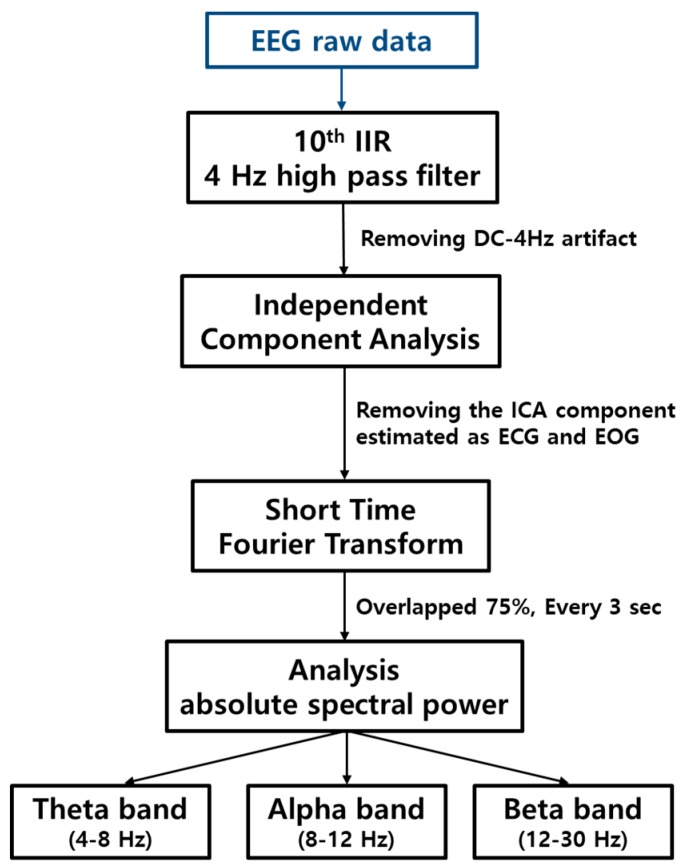
The block diagram of the signal processing where IIR represents infinite impulse response.

Figure 1 shows the block diagram of signal processing. There are two parts: filtering and removing artifacts, and analysis in the frequency domain.

(1) $$Y\left(n\right)=-\sum A_{k}Y\left(n-k\right)+\sum B_{k}X\left(n-k\right)$$

**Figure 2:**
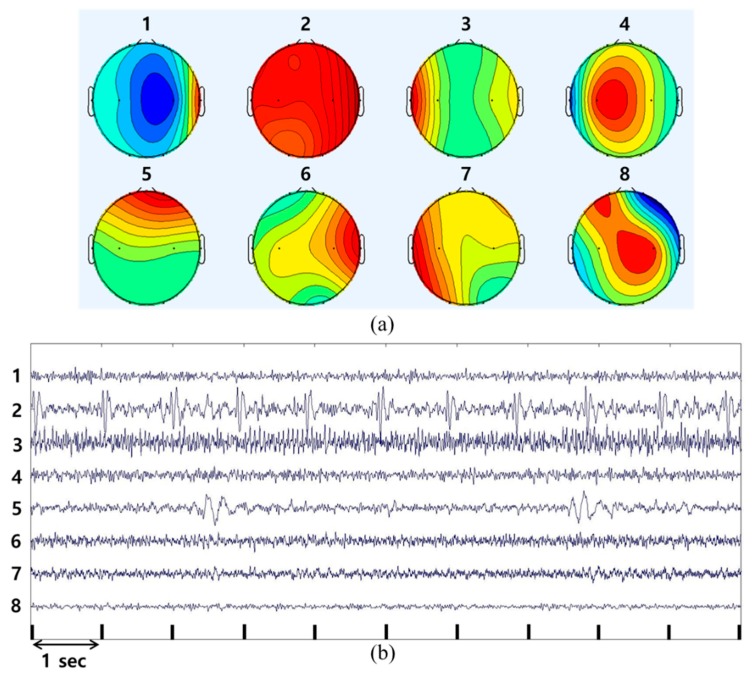
Identification of interfering signals-based an independent component analysis: (**a**) Topography of the eight ICA components; (**b**) waveforms of the eight ICA components with respect to time.

It is the Infinite Impulse Response (IIR) digital filter formula shown in the above [23] where X(n) is the input of the filter, Y(n) is the output of the filter, and A_k_, B_k_ is the *k*th order filter coefficient. In this study, a 10th order IIR digital high-pass filter with a cutoff frequency of 4 Hz was applied to raw data before calculating EEG results, and then, unnecessary signals were removed through ICA. The digital filter was designed using the Filter Design and Analysis (FDA) tool, which is one of the signal processing toolboxes provided by MATLAB^TM^. ICA is a method of separating several independent components contained in a signal, and it is used to extract the characteristics of a signal or remove noise. ICA can be used to detect the location of a specific brain signal in EEG results and to remove mixed noise in EEG signals [24]. Figure 2 shows the results obtained using EEGLAB, which is one of the open-source toolboxes developed for MATLAB^TM^ by the Swartz Center for Computational Neuroscience (SCCN) in San Diego, US [25].

**Figure 3:**
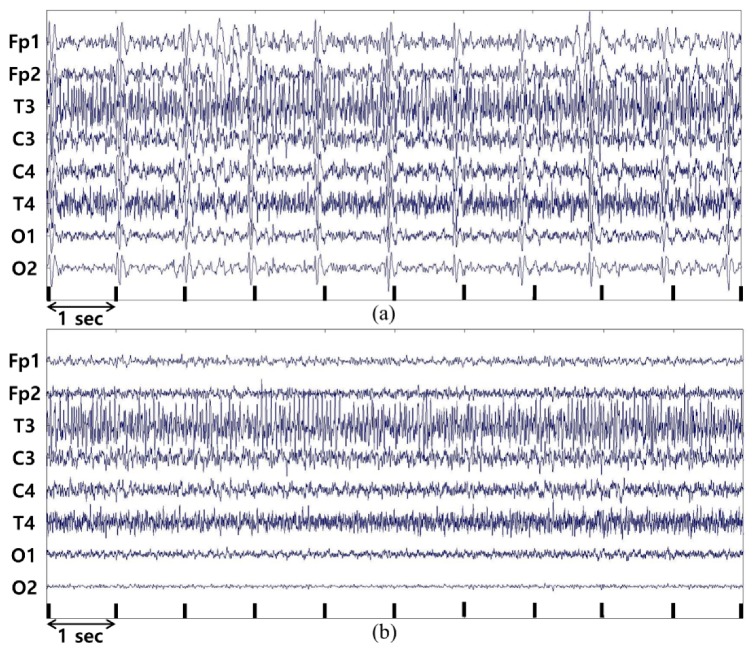
(**a**) Original EEG data in each channel; (**b**) Processed data after removal of ECG and EOG related ICA components.

Each ICA component was checked to remove EOG and ECG noise. The process of removing EOG and ECG noise using ICA is as follows: Figure 2a shows the topography of the 8 ICA components. Component numbers are horizontally assigned from the top left. In Figure 2b, the ICA components are sequentially displayed on the time axis. The component numbers in both figures correspond to each other. For example, Component 1 in Figure 2a corresponds to Component 1 in Figure 2b. Therefore, the location of the signal is determined in Figure 2a, and its real-time waveform is shown in Figure 2b. The ECG and EOG components could be estimated by checking the signal pattern. The ECG signal appears along the cardiac cycle in the entire area of the brain. The second ICA component is similar to the ECG signal and appears to affect the entire area of the brain. It has a period of approximately one second, which corresponds to a heart rate of approximately 60 bpm and falls within the normal heart rate range for an adult. Therefore, the second ICA component represents the ECG signal. The EOG signal primarily appears in Fp1 and Fp2, with large signal amplitude. The fifth ICA component is shown by the frequency of large amplitudes in Fp1 and Fp2 in Figure 3a. This frontal lobe is clearly reflected in the EOG signal; hence, the fifth ICA component in Figure 2 can be considered as the EOG signal. Therefore, if the 2nd and 5th ICA components are removed, the interference components due to the ECG and EOG signals can be excluded.

Figure 3 shows a comparison of the original EEG data and the data after removing interference components through ICA. Figure 3a shows the original data and Figure 3b shows the waveforms after the data are processed using the ICA method. Removing the ICA components estimated as ECG and EOG signals shows that the periodic components included in the original data have been removed. Spectral analysis was performed to obtain the results from the data collected after noise removal. After noise removal, Short-Time Fourier Transform (STFT) is adopted to analysis the data that is transformed as overlapped 75% every 3 s. For splitting each band, the absolute spectral power data is calculated from those results. As this study is aimed at normal subjects in a state of arousal, the signals in Theta, Alpha, and Beta waves were analyzed, and Delta and Gamma waves were excluded.

## 3. Results

**Figure 4:**
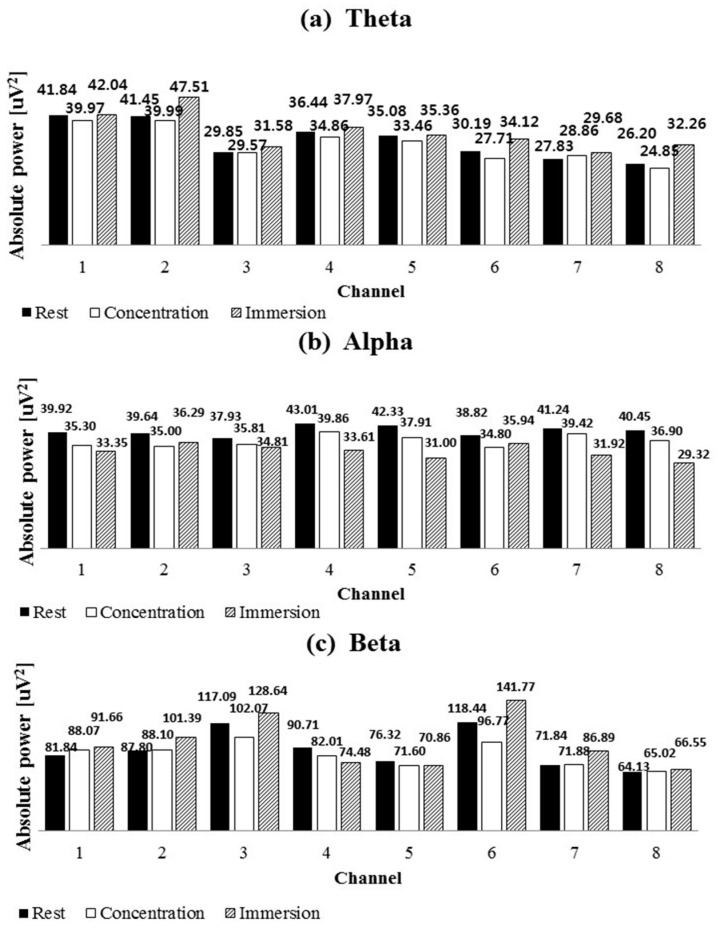
The absolute powers of Theta (**a**); Alpha (**b**) and Beta (**c**) bands are shown in the cases of rest, concentration and immersion.

**Figure 5:**
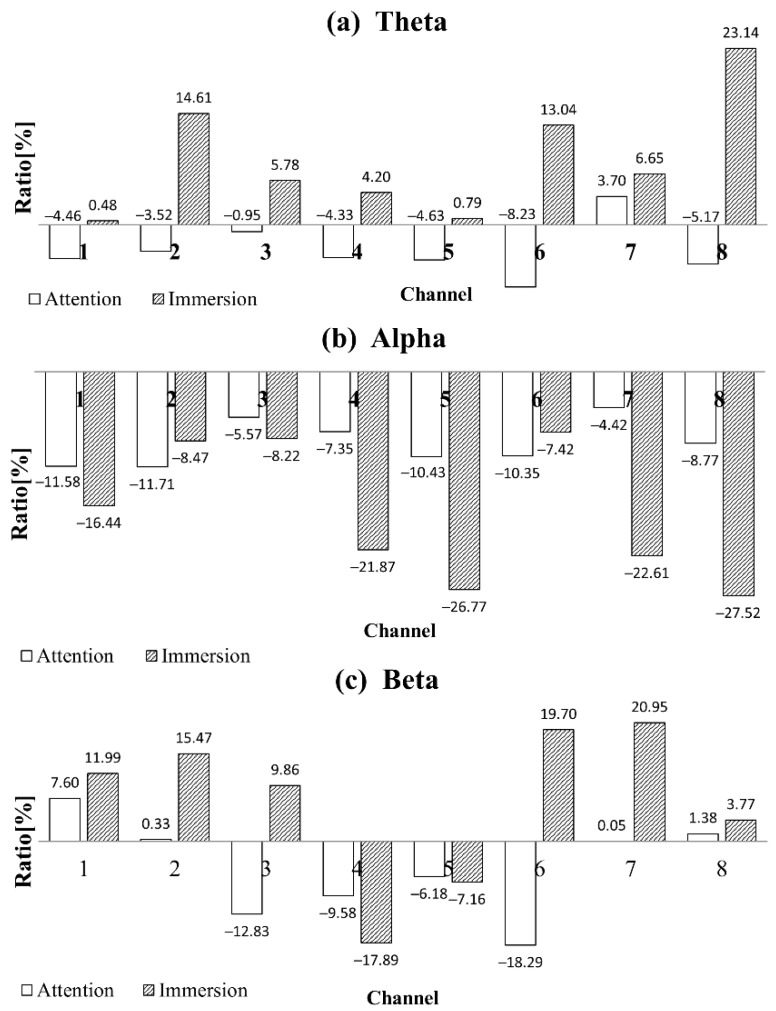
The absolute power ratio with respect to that of rest state where the two cases of concentration and immersion states are shown in the frequency bands of Theta (**a**); Alpha (**b**) and Beta (**c**).

Figure 4 shows the Fourier transformation result for the mean absolute power of the Theta, Alpha, and Beta bands of the 32 subjects for each channel. It shows the values for the rest, concentration, and immersion states of each band. Figure 5 shows the ratios of the absolute powers for the concentration and immersion states to that for the rest state. There are rest, concentration, and immersion states for each band, and the mean absolute power of the 32 subjects in each channel is compared between the concentration and immersion states and the rest state. 

Figure 5 clearly shows how change occurs compared to the rest state. As shown by the results of Channels 1 and 2 in the Theta band, a decrease of 4.46% and 3.52% is observed in the concentration state, while an increase of 0.48% and 14.61% is observed in the immersion state, relative to the rest state. In addition, Channels 7 and 8 of the Theta band show a larger increase of 6.65% and 23.14% in the immersion state, as compared to an increase of 3.70% and 5.17% in the concentration state. In the immersion state, the Theta band increases by 0.48–23.14% in all channels; however, in the concentration state, it decreases in almost all channels. The Alpha band decreases in the concentration and immersion states compared to the rest state; however, the decrease in the immersion state is more pronounced. The decrease in the concentration state is 4.42–11.71% while that in the immersion state is 7.42–27.52%. Particularly, in the immersion state, Channels 7 and 8 show the highest decrease of 22.61% and 27.52%. The Beta band increases in the concentration and immersion states at Channels 1, 2, 7, and 8; it increases by 3.77–20.95% in the immersion state and by 0.05–7.60% in the concentration state. In the Beta band, the temporal lobes of Channels 3 and 6 decrease in the concentration state compared to the rest state; however, they increased in the immersion state. In the Beta band, the central lobe for Channels 4 and 5 decreases in the concentration and immersion states compared to the rest state.

**Figure 6:**
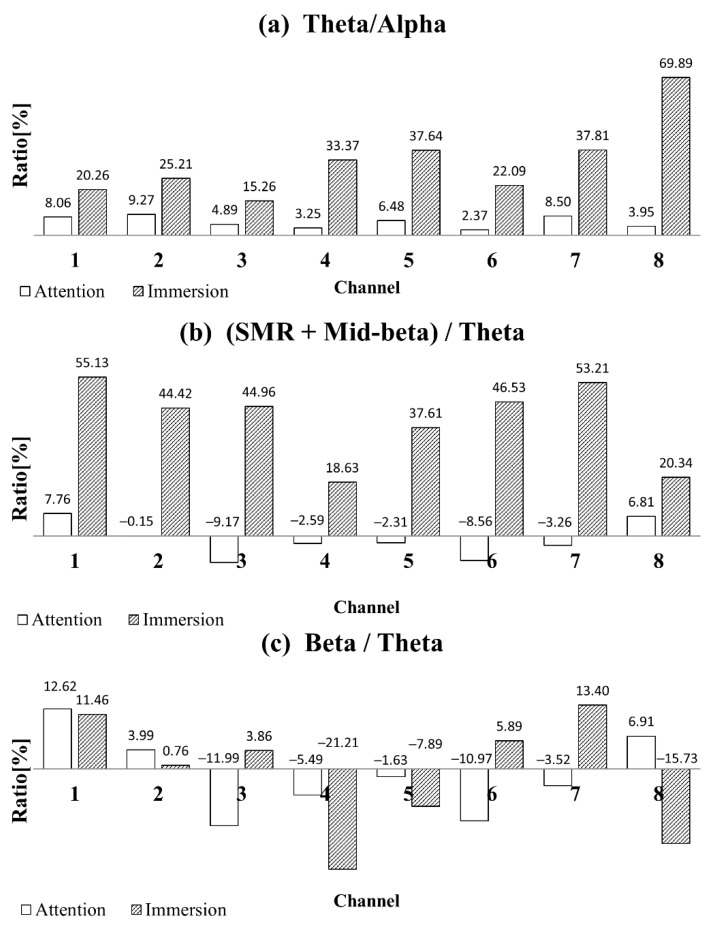
Three different indices in each channel are compared in the cases of concentration and immersion.

Various parameters (also referred to as the index) have been proposed to represent the degree of concentration or immersion using a single number. For example, this study used the ratio of the Theta and Alpha bands as an index to represent immersion. In a previous study, the ratio of the sum of the SMR and Mid-B bands to the Theta band was used as an index for concentration [18]. Figure 6 shows the increase/decrease ratio of the concentration and immersion states relative to the rest state for three indices. The concentration and immersion states were compared to the rest state as the bases by applying each index to rest, concentration, and immersion data.

In the case where Theta/Alpha is used as the immersion index, the change in this value for concentration or immersion relative to rest is considered. In the immersion state, the value increases by 15.26–69.89% in all channels, particularly, 37.81% and 69.89% in Channels 7 and 8, respectively. This indicates that the occipital lobe channel is highly active in the immersion state. In addition, the value increases in all channels during concentration; however, it increases only slightly from 2.37% to 9.27% compared to the immersion state. The value of Theta/Alpha appears to be appropriate as an index for immersion.

In the case where (SMR + Mid-B)/Theta is used as the concentration index, considering the concentration state relative to the rest state, this concentration index increases by 7.76% or decreases by 0.15% in Channels 1 and 2. Compared to the studies by Ga et al. [18], in which the attention level in the frontal lobe is elevated, this result does not explain the concentration state clearly. Rather, when immersion is determined using this concentration index, the phenomenon becomes more difficult to explain, as the index is larger during immersion than during concentration.

The concentration index proposed in this study is Beta/Theta. The results for concentration and immersion represented using this index are shown in Figure 6c. When the ratio of the Beta band to the Theta band is used as an index for concentration, the values for Channels 1 and 2 clearly increase by 12.62 and 3.99% in the concentration state, relative to the rest state. The values in the immersion state increase by 18.63–55.13% in all channels when (SMR + Mid-B)/Theta is used as the concentration index. When Beta/Theta is used as the concentration index, the values decrease by 7.89–21.21% in Channels 4, 5, and 8 or increase by 0.76–13.40% in Channels 1, 2, 3, 6, and 7. A distinctive pattern is observed. In other words, the results are inconsistent when the concentration index is applied to determine the immersion state. This can be advantageous as this is a concentration index that is not affected by immersion. The index was applied to Channels 1, 2, 7, and 8 to determine immersion. When immersion is evaluated using the immersion index, it is observed to increase in all channels as shown in Figure 6; however, the increase is higher in Channels 7 and 8 in the occipital lobe.

Table 1 shows the results obtained after applying the immersion index to Channels 7 and 8 in the occipital lobe and Channels 1 and 2 in the frontal lobe. This table shows ratio of the values for the occipital and frontal lobes and the ratio of increase/decrease in concentration and immersion relative to the rest state. Relative to the rest state, a decrease of 2.20% is observed in the concentration state and an increase of 25.09% is observed in the immersion state. This implies the increase/decrease ratio in Channels 7 and 8 decreases more than that in Channels 1 and 2 in the concentration state when the immersion index is applied and the increase/decrease ratio in Channels 7 and 8 increases in the immersion state.

All statistical tests were conducted with SPSS version 24. Wilcoxon signed-ranking test was used to analyze the effectiveness of EEG index for comparison between concentration and immersion state. The results are shown in Table 2. Table 2 shows that the two immersion indices (Theta/Alpha, and [Theta/Alpha (7, 8)]/[Theta/Alpha (1, 2)]) are significantly higher in the immersion state compare to the concentration state. In case of the concentration indices ((SMR+Mid-β)/Theta, and Beta/Theta), there are statistically significant differences between concentration and immersion in Channels 1, 2, 4, 5 and 8. However, the (SMR+Mid-β)/Theta is higher in immersion state, and on the other hand, the Beta/Theta is higher in concentration state. Therefore, the concentration and immersion states can be distinguished using a new immersion index for Channels 1 and 2 and Channels 7 and 8.

## 4. Discussion

Interfering signals and noise should be processed to analyze brain waves. For this purpose, signal processing was performed based on the Infinite Impulse Response filter and the ICA. The Delta and Gamma bands were excluded from analysis as the study did not involve patients with sleeping or brain diseases.

In the absolute power analysis, the Alpha band in the concentration and immersion states decreases as compared to the rest state in all channels (Figure 4). The decrease is more pronounced in the immersion state. Such a result is consistent with that of a previous study, which is that Alpha waves are typically best observed at rest and decrease when stimuli are applied. The Beta band increase in the concentration and immersion states in Channels 1, 2, 7, and 8; the increase in the immersion state is higher. In general, Beta waves appear irregularly and are small in size. They typically appear in an awakened or an active state or in a state of thought and concentration. They are symmetrically distributed throughout the brain and appear clearly at the front of the brain (frontal lobe) [26]. Beta wave activity is observed in exercise-related cortex or basal ganglia while moving or concentrating on hands and feet. This explains the increase in Beta waves during immersion in the computer game or during concentration in this study. The Beta band decreases in the concentration state in Channels 3 and 6; however, it increases significantly in the immersion state. This phenomenon may be limited to playing the computer game in this study. A hearing response is observed in temporal lobes as the activity of the brain waves increases because subjects can listen to the sound of the computer game. The Beta bands in Channels 4 and 5 of the central lobe decrease in the concentration and immersion states, and the decrease is considerably larger in the immersion state. 

The characteristics of concentration and immersion in the Theta band are different. Theta waves are observed in various parts of the brain; Theta waves are either found or not found in cognitive activity and they are observed in morbid conditions. They are known to have several different generation principles. Among various cognitive activities, Theta waves are typically observed in the central frontal cortex, and they vary in magnitude according to the intensity of the memorizing activity while being independent of Alpha waves. In almost all channels, the absolute power in the concentration state decreases more than that in the rest state (Figure 4). However, it increases significantly in the immersion state in almost all channels. The similarity between the concentration and immersion states in the frontal lobe is the increase in the Beta band and the general decrease in the Alpha band. The Theta band decreases in frontal lobe during concentration and increases during immersion.

Concentration and immersion show significant difference in the Theta, Alpha and Beta bands. Based on the results of the Beta band activation in the frontal lobe during concentration, it can be inferred that the immersion state is similar to the concentration state. However, the differences in the Theta band can distinguish between the two states. In the immersion state, it is possible to infer Theta band activation from the phenomenon of immersing in the game and forgetting the surrounding environment and problems; this is a state similar to conscious sleep. On the contrary, as concentration is a selective phenomenon (between the white background and red dot), it is different from immersion, as shown by the decrease in the Theta band. Therefore, the ratio of Theta band to the Alpha band is an appropriate index to distinguish the immersion state from the concentration state.

When the concentration and immersion states are compared using the immersion index (Theta/Alpha) (Figure 6a), the index is observed to increase in all channels during immersion, particularly in Channels 7 and 8. Moreover, it increases in all channels during concentration; however, the increase is considerably smaller than in the immersion state. When analysis is performed using the ratio of the average in Channels 1 and 2 and that in Channels 7 and 8 in the immersion index, the ratio in the concentration state decreases as compared to the rest state. However, the ratio increases in the immersion state. Therefore, the selection of a particular channel in the immersion index can provide a new index that can distinguish between the concentration and immersion states. In the concentration state, when data are analyzed using the concentration index as the ratio of the sum of SMR and Mid-B band to the Theta band (Figure 6b), relative to the resting state, the index increases in Channel 1 and decreases in Channel 2 in the concentration state, making it difficult to clearly observe the increase in concentration level in the frontal lobe.

In the cases where the concentration index is set as the ratio of the entire Beta band, including high-Beta, to the Theta band (Figure 6c), the increase in Channels 1 and 2 is considerably clear in the concentration state, which implies that it is more desirable to use "the ratio of the Beta and Theta bands" as an index for concentration. In addition, the immersion state represented using the Beta/Theta concentration index does not exhibit a consistent pattern, as shown by either an increase in all channels or a different increase/decrease in each channel. This concentration index does not clearly correlate with the immersion state, and it selectively shows the concentration state rather than the immersion state; therefore, it is more preferred as a concentration index.

The difference between the two indices, i.e., (SMR + Mid-B)/Theta and Beta/Theta, lies in the high-Beta band of 20–30 Hz. The high-Beta band is commonly associated with stress. Unlike the rest state, brain waves are recorded using the activity of staring at a red dot on a white screen to measure the concentration state. Unlike the rest state, measuring the concentration state can cause stress because of the changed experimental environment. If so, it can be questioned whether the high-Beta band should be excluded from the concentration indicators proposed in previous studies. The concentration index in previous studies is the ratio of the sum of the SMR and Mid-B bands to the Theta band. Among the studies that have used this index, there is a study that cites the contents of an EEG measurement instrument manual. However, there is no ground test or publications on the indicators cited in the EEG manuals; thus, the use of concentration indicators from previous studies is considered to be a weak basis. The experimental results show that the ratio of the Beta band to the Theta band is more optimal as a concentration index.

## 5. Conclusions

In this study, the mental states of concentration and immersion whose characteristics are similar could be distinguished through EEG analysis. Both concentration and immersion states increased Alpha waves. However, a difference was observed with Theta waves where Theta waves decreased during concentration and, on the other hand, increased during immersion. With Beta waves, differences between concentration and immersion show much more complicated patterns depending also on brain regions. The concentration index in previous studies was the ratio of the sum of the SMR and Mid-B bands to the Theta band. In this study, we find that the concentration index of Beta/Theta was more promising. We also propose a new index of indicating concentration or immersion that is the ratio of Theta-Alpha ratio between occipital lobe and frontal lobes. Relative to the rest state, this new index was decreased in the concentration state and increased in the immersion state. Considering all these, an apparent difference between concentration and immersion could be measured. This implies that there are possibilities of diagnosing various mental states other than concentration and immersion and that more elaborate control in brain-machine interface may be possible in the future.

## Figures and Tables

**Table 1:** The Theta-Alpha ratio between Channels 7 and 8 and Channels 1 and 2 as an immersion index.

Index	State	Value	Ratio	Value (%)
[Theta/Alpha (7, 8)]	Rest	0.63	Concentration/Rest	−2.20
	Concentration	0.62		
[Theta/Alpha (1,2)]	Immersion	0.79	Immersion/Rest	25.09

**Table 2:** Comparison between concentration and immersion state using four indices.

Index	Channel	Concentration	Immersion	*p* Value
(SMR + Mid-β)/Theta	1	0.92	1.33	0.036
	2	0.91	1.31	0.029
	3	1.50	2.39	0.881
	4	1.06	1.30	0.040
	5	1.01	1.42	0.018
	6	1.48	2.37	0.477
	7	1.17	1.85	0.489
	8	1.24	1.40	0.001
Beta/Theta	1	2.20	2.18	0.052
	2	2.20	2.13	0.210
	3	3.45	4.07	0.432
	4	2.35	1.96	0.043
	5	2.14	2.00	0.045
	6	3.49	4.16	0.270
	7	2.49	2.93	0.852
	8	2.62	2.06	0.001
Theta/Alpha	1	1.13	1.26	0.003
	2	1.14	1.31	0.004
	3	0.83	0.91	0.009
	4	0.87	1.13	<0.001
	5	0.88	1.14	<0.001
	6	0.80	0.95	<0.001
	7	0.73	0.93	<0.001
	8	0.67	1.10	<0.001
[Theta/Alpha (7, 8)]/[Theta/Alpha (1,2)]	-	0.62	0.79	0.004
